# A modified nonmonotone BFGS algorithm for unconstrained optimization

**DOI:** 10.1186/s13660-017-1453-5

**Published:** 2017-08-09

**Authors:** Xiangrong Li, Bopeng Wang, Wujie Hu

**Affiliations:** Guangxi Colleges and Universities Key Laboratory of Mathematics and Its Applications, College of Mathematics and Information Science, Guangxi University, Nanning, Guangxi P.R. China

**Keywords:** 65K05, 90C26, BFGS update, global convergence, superlinear convergence, nonmonotone

## Abstract

In this paper, a modified BFGS algorithm is proposed for unconstrained optimization. The proposed algorithm has the following properties: (i) a nonmonotone line search technique is used to obtain the step size $\alpha_{k}$ to improve the effectiveness of the algorithm; (ii) the algorithm possesses not only global convergence but also superlinear convergence for generally convex functions; (iii) the algorithm produces better numerical results than those of the normal BFGS method.

## Introduction

Consider

1.1 $$ \min \bigl\{ f(x) \vert x \in \Re^{n} \bigr\} , $$

where $f(x):\Re^{n}\rightarrow \Re $ is continuously differentiable. Many similar problems can be transformed into the above optimization problem (see [1–16] etc.). The following iteration formula is used to address the iteration point of (1.1):

1.2 $$ x_{k+1}=x_{k}+\alpha_{k}d_{k},\quad k=0, 1, 2,\ldots, $$

where $x_{k}$ is the *k*th iterative point, $\alpha_{k}>0$ is the step length, and $d_{k}$ is the search direction of *f* at $x_{k}$. The search direction $d_{k}$ determines the line search method (see [17–25]). The quasi-Newton method is defined by

1.3 $$ B_{k}d_{k}+g_{k}=0, $$

where $g_{k}=\nabla f(x_{k})$, $B_{k}$ is the quasi-Newton update matrix, and the sequence $\{B_{k}\}$ satisfies the so-called quasi-Newton equation

1.4 $$ B_{k+1}s_{k}=y_{k}, $$

where $s_{k}=x_{k+1}-x_{k}$, $y_{k}=g_{k+1}-g_{k}$, and $g_{k+1}= \nabla f(x_{k+1})$. The following update of $B_{k}$:

1.5 $$ B_{k+1}=B_{k}-\frac{B_{k}s_{k}s_{k}^{T}B_{k}}{s_{k}^{T}B_{k}s_{k}}+ \frac{y _{k}y_{k}^{T}}{s_{k}^{T}y_{k}} $$

is the BFGS formula (Broyden [26], Fletcher [27], Goldfar [28], and Shanno [29]), which is one of the most effective quasi-Newton methods. Convex functions can be combined with exact line or certain special inexact line search techniques that have global convergence (see [30–32] etc.) and superlinear convergence (see [33, 34] etc.). For general functions, under inexact line search techniques, Dai [35] constructed an example to show that the BFGS method fails. Mascarenhas [36] proved the nonconvergence of this method, even with the exact line search technique. To obtain global convergence of a BFGS method without the convexity assumption, Li and Fukushima [37, 38] proposed the following modified BFGS methods.

### Formula 1

[37]

The BFGS update formula is defined by

1.6 $$ B_{k+1}=B_{k}+\frac{\delta_{k}^{T}\delta_{k}}{s_{k}^{T}\delta_{k}}- \frac{B _{k}s_{k}s_{k}^{T}B_{k}}{s_{k}^{T}B_{k}s_{k}}, $$

where $\delta_{k}=y_{k}+(\max \{0,-\frac{y_{k}^{T}s_{k}}{\Vert s_{k}\Vert ^{2}}\}+\phi (\Vert g_{k}\Vert ))s_{k}$ and function $\phi:\Re \rightarrow \Re $ satisfies: (i) $\phi (t)>0$ for all $t>0$; (ii) $\phi (t)=0$ if and only if $t=0$; (iii) if *t* is in a bounded set, and $\phi (t)$ is bounded. Using the definition of $\delta_{k}$, it is not difficult to obtain

$$\delta_{k}^{T}s_{k}\geq \max \bigl\{ s_{k}^{T}y_{k},\phi \bigl(\Vert g_{k}\Vert \bigr)\Vert s_{k}\Vert ^{2}\bigr\} >0. $$

This is sufficient to guarantee the positive definiteness of $B_{k+1}$ as long as $B_{k}$ is positive definite. Li and Fukashima presented $\phi (t)=\mu t$ with some constant $\mu >0$.

### Formula 2

[38]

The BFGS update formula is defined by

1.7 $$\begin{aligned} B_{k+1}=\textstyle\begin{cases} B_{k}+\frac{\delta_{k}^{T}\delta_{k}}{s_{k}^{T}\delta_{k}}-\frac{B _{k}s_{k}s_{k}^{T}B_{k}}{s_{k}^{T}B_{k}s_{k}}, & \mbox{if } \frac{\delta_{k}^{T}s_{k}}{\Vert s_{k}\Vert ^{2}}\geq \phi (\Vert g_{k}\Vert ), \\ B_{k},& \mbox{otherwise}, \end{cases}\displaystyle \end{aligned}$$

where $\delta_{k}$, *ϕ* and the properties are the same as those in Formula 1. For nonconvex functions, these two methods possess global convergence and superlinear convergence.

Some scholars have conducted further research to obtain a better approximation of the Hessian matrix of the objective function.

### Formula 3

[39]

The BFGS update formula is defined by

1.8 $$ B_{k+1}=B_{k}-\frac{B_{k}s_{k}s_{k}^{T}B_{k}}{s_{k}^{T}B_{k}s_{k}}+ \frac{y _{k}^{m*}{y_{k}^{m*}}^{T}}{s_{k}^{T}y_{k}^{m*}}, $$

where $y_{k}^{m*}=y_{k} + \frac{\rho_{k}}{\Vert s_{k}\Vert ^{2}}s_{k}$ and $\rho_{k}=2[f(x_{k})-f(x_{k}+\alpha_{k}d_{k})]+(g(x_{k}+\alpha_{k} d _{k})+g(x_{k}))^{T}s_{k} $. It is easy to conclude that this formula contains both gradient and function value information. One may believe that the resulting methods will outperform the normal BFGS method. In fact, the practical computation shows that the method is better than the normal BFGS method and that it has some theoretical advantages (see [39, 40]). Under the WWP line search, Wei *et al.* [39] proposed the quasi-Newton method and established its superlinear convergence for uniformly convex functions. Its global convergence can be found in [40], but the method fails for general convex functions. One of the main reasons for the failure is the non-positive definiteness of matrix $B_{k}$ for general convex functions. Byrd *et al.* [31, 32] showed that the positive definiteness of matrix $B_{k}$ plays an important role in the convergence of the quasi-Newton algorithm. Yuan and Wei [41] first analyzed the global convergence and superlinear convergence of the modified BFGS formula in [39] using gradient and function value information for general convex functions. Based on equation (1.9), Yuan and Wei [41] proposed another BFGS formula.

### Formula 4

[41]

The BFGS update formula is defined by

1.9 $$ B_{k+1}=B_{k}-\frac{B_{k}s_{k}s_{k}^{T}B_{k}}{s_{k}^{T}B_{k}s_{k}}+ \frac{y _{k}^{m}{y_{k}^{m}}^{T}}{s_{k}^{T}y_{k}^{m}}, $$

where $y_{k}^{m}=y_{k} + \max \{\frac{\rho_{k}}{\Vert s_{k}\Vert ^{2}},0\}s _{k}$. This modified method obtains global convergence and superlinear convergence for generally convex functions. The same work was previously performed by Zhang *et al.* [42].

### Formula 5

[42]

The BFGS update formula is defined by

1.10 $$ B_{k+1}=B_{k}-\frac{B_{k}s_{k}s_{k}^{T}B_{k}}{s_{k}^{T}B_{k}s_{k}}+ \frac{y _{k}^{1*}{y_{k}^{1*}}^{T}}{s_{k}^{T}y_{k}^{1*}}, $$

where $y_{k}^{1*}=y_{k}+\bar{A}_{k}s_{k}$, $\bar{A}_{k}=\frac{6[f(x _{k})-f(x_{k}+\alpha_{k}d_{k})]+3(\nabla f(x_{k}+\alpha_{k} d_{k})+ \nabla f(x_{k}))^{T}s_{k}}{\Vert s_{k}\Vert ^{2}}$. It is clear that the quasi-Newton equation (1.10) also contains both gradient and function value information, and it has been proved that the new formula has a higher order approximation to $\nabla^{2} f(x)$. Furthermore, Yuan *et al.* [43] extended a similar technique to $y_{k}^{1*}$ in a limited memory BFGS method, where global convergence is only obtained for uniformly convex functions. Several other modified quasi-Newton methods have been reported (see [23, 40, 44, 45]).

The monotone line search technique is often used to determine the step size $\alpha_{k}$. One famous technique is the weak Wolfe-Powell (WWP) technique.

(i) *WWP line search technique*. $\alpha_{k}$ is determined by
  1.11 $$ f(x_{k}+\alpha_{k}d_{k})\leq f(x_{k})+\delta \alpha_{k}g_{k}^{T}d_{k},\qquad g(x_{k}+\alpha_{k}d_{k})^{T}d_{k} \geq \sigma g_{k}^{T}d_{k}, $$
  where $0<\delta <\sigma <1$. Recently, a modified WWP line search technique was proposed by Yuan, Wei, and Lu [46] to ensure that the BFGS and the PRP methods have global convergence for nonconvex functions; these two open problems have been solved. However, monotonicity may generate a series of extremely small steps if the contours of the objective functions are a family of curves with large curvature [47]. Nonmonotonic line search to solve unconstrained optimization was proposed by Grippo *et al.* in [47–49] and was further studied by [50]. Grippo, Lamparillo, and Lucidi [47] proposed the following nonmonotone line search and called it GLL line search.
(ii) *GLL nonmonotone line search*. $\alpha_{k}$ is determined by
  1.12 $$\begin{aligned}& f(x_{k+1}) \leq \max_{0\leq j \leq M_{0}}f(x_{k-j})+ \epsilon_{1}\alpha _{k}g_{k}^{T}d_{k}, \end{aligned}$$
  1.13 $$\begin{aligned}& g(x_{k+1})^{T}d_{k} \geq \max \bigl\{ \epsilon_{2}, 1-\bigl(\alpha_{k}\Vert d_{k} \Vert \bigr)^{p} \bigr\} g_{k}^{T}d_{k}, \end{aligned}$$
  where $p\in (-\infty,1)$, $k=0, 1, 2, \ldots$ , $\varepsilon_{1} \in (0,1)$, $\varepsilon_{2} \in (0,\frac{1}{2})$, $M_{0}$ is a nonnegative integer. By combining this line search with the normal BFGS formula, Han and Liu [51] established the global convergence of the convex objective function; its superlinear convergence was established by Yuan and Wei [52]. Although these nonmonotone techniques perform well in many cases, the numerical performance is dependent on the choice of $M_{0}$ to some extent (see [47, 53, 54] in detail). Zhang and Hager [55] presented another nonmonotone line search technique.
(iii) *Zhang and Hager nonmonotone line search technique* [55]. In this technique $\alpha_{k}$ is found by
  1.14 $$ Q_{k+1}=\eta_{k}Q_{k}+1,\qquad C_{k+1}= \frac{\eta_{k}Q_{k}C_{k}+f(x_{k+1})}{Q_{k+1}}, $$
  where $\eta_{k}\in [\eta_{\min },\eta_{\max }]$, $0\leq \eta_{\min } \leq \eta_{\max }\leq 1$, $C_{0}=f(x_{0})$ and $Q_{0}=1$. It is easy to conclude that $C_{k+1}$ is a convex combination of $C_{k}$ and $f(x_{k+1})$. The numerical results show that this technique is more competitive than the nonmonotone method of [47], but it requires strong assumption conditions for convergence analysis.

Motivated by the above observations, we study the modified BFGS-type method of Yuan *et al.* [43] based on the formula (1.10). The modified BFGS-type method and the proposed algorithm have the following characteristics:

- The GLL line search technique is used in the algorithm to ensure good convergence.
- The major contribution of the new algorithm is an extension of the modified BFGS update from [43] and [42].
- Another contribution is the proof of global convergence for generally convex functions.
- The major aim of the proposed method is to establish the superlinear convergence and the global convergence for generally convex functions.
- The experimental problems, including both normal unconstrained optimization and engineering problems (benchmark problems), indicate that the proposed algorithm is competitive with the normal method.

This paper is organized as follows. In the next section, we present the algorithm. The global convergence and superlinear convergence are established in Section 3 and Section 4, respectively. Numerical results are reported in Section 5. In the final section, we present a conclusion. Throughout this paper, $\Vert \cdot \Vert $ denotes the Euclidean norm of a vector or matrix.

## Algorithm

In this paper, we study the modified formula of [43] and obtain global convergence and superlinear convergence under generally convex conditions. The modified BFGS update of (1.10) is presented as

2.1 $$ B_{k+1}^{*}=B_{k}^{*} - \frac{B_{k}^{*} s_{k} s_{k}^{T} B_{k}^{*}}{s _{k}^{T} B_{k}^{*} s_{k}} + \frac{y_{k}^{*} {y_{k}^{*}}^{T}}{{y_{k} ^{*}}^{T} s_{k}}, $$

where ${y_{k}^{*}}=y_{k}+A_{k}^{*}s_{k}$, $A_{k}^{*}=\max \{\bar{A} _{k},0\}$. The corresponding quasi-Newton equation is

2.2 $$ B_{k+1}^{*}s_{k}=y_{k}^{*}. $$

By the definition of the convex property of *f*, $s_{k}^{T}y_{k}^{*}>0$ holds (see [43] in detail). Therefore, the update matrix $B_{k+1}^{*}$ from (2.1) inherits the positive definiteness of $B_{k}^{*}$ for generally convex functions. Now, we state the algorithm as follows.

### Algorithm 1

Mod-non-BFGS-A

Step 0: Given a symmetric and positive definite matrix $B_{0}^{*}$ and an integer $M_{0}>0$, choose an initial point $x_{0} \in \Re^{n}$, $0<\varepsilon <1$, $0<\epsilon_{1}<\epsilon_{2}<1$, $p\in (-\infty,1)$; Set $k:=0$.
Step 1: $\Vert g_{k}\Vert \leq \varepsilon$, stop; Otherwise, go to the next step.
Step 2: Solve
  2.3 $$ B_{k}^{*}d_{k}+g_{k}=0 $$
  to obtain $d_{k}$.
Step 3: The step length $\alpha_{k}$ is determined by GLL (1.12) and (1.13).
Step 4: Let $x_{k+1}=x_{k}+\alpha_{k}d_{k}$.
Step 5: Generate $B_{k+1}^{*}$ from (2.1) and set $k=k+1$; Go to Step 1.

## Global convergence

The following assumptions are required to obtain the global convergence of Algorithm 1.

### Assumption A

(i) *The level set* $\L_{0}=\{x \mid f(x) \le f(x _{0}) \}$ *is bounded*.
(ii) *The objective function* *f* *is continuously differentiable and convex on* $L_{0}$. *Moreover*, *there exists a constant* $L\ge 0$ *satisfying*
  3.1 $$ \bigl\Vert g(x)-g(y)\bigr\Vert \le L\Vert x-y \Vert ,\quad \forall x, y \in L_{0}. $$

Assumption A implies that there exist constants $M>0$ and $\varrho >0$ satisfying

$$\bigl\Vert G(x)\bigr\Vert \leq M,\qquad G(x)=\nabla^{2} f(x), \quad x\in L_{0}, $$

and

3.2 $$ \frac{\Vert y_{k}\Vert ^{2}}{s_{k}^{T}y_{k}}\leq \varrho,\quad k\geq 0\ (\mbox{see [56]}). $$

### Lemma 3.1

*Suppose Assumption* A *holds*. *Then there exists a constant* $M_{*}>0$ *such that*

$$\frac{\Vert y^{*}_{k}\Vert ^{2}}{s^{T}_{k}y^{*}_{k}}\leq M_{*}. $$

The proof is similar to [41], so it is not presented here.

### Lemma 3.2

*Let* $B_{k}$ *be updated by* (2.1); *then the relation*

$$\det \bigl(B_{k+1}^{*}\bigr)=\det \bigl(B_{k}^{*} \bigr)\frac{(y_{k}^{*})^{T} s_{k}}{s_{k} ^{T} B_{k}^{*}s_{k}} $$

*holds*, *where* $\det (B_{k}^{*})$ *denotes the determinant of* $B_{k}^{*}$.

### Lemma 3.3

*Assume that Assumption* A *holds and that sequence* $\{x_{k}\}$ *is generated by Algorithm* 1. *If*

$$\liminf_{k\rightarrow \infty } \Vert g_{k}\Vert >0, $$

*then there exists a constant* $\epsilon '>0$ *satisfying*

$$\prod_{j=1}^{k} \gamma_{j} \geq \bigl(\epsilon '\bigr)^{k},\quad \textit{for all }k\geq 1, $$

*where* $\gamma_{j}=\frac{-g_{j}^{T}d_{j}}{\Vert d_{j}\Vert }$.

### Proof

For $k=0$, by the positive definiteness of $B_{0}$, we have $s_{0}^{T}y_{0}^{*}>0$. Then $B_{1}$ is generated by (2.1), and $B_{1}$ is positive definite. Assume that $B_{k}$ is positive definite; for all $k\geq 1$, we prove that $s_{k}^{T}y_{k}^{*}>0$ holds by the following three cases.

*Case 1:* $\bar{A}_{k}<0$ *.* The definition of $y_{k}^{*}$, the convexity of $f(x)$, and Assumption A generate

$$s_{k}^{T}y_{k}^{*}=s_{k}^{T}y_{k}>0. $$

*Case 2:* $\bar{A}_{k}=0$ *.* By (1.13), (2.3), Assumption A, the definition of $y_{k}^{*}$, and the positive definiteness of $B_{k}$, we get

$$s_{k}^{T}y_{k}^{*}=s_{k}^{T}y_{k} \geq -(1-\sigma_{*})\alpha_{k}d_{k} ^{T}g_{k}=(1-\sigma_{*})\alpha_{k}d_{k}^{T}B_{k}^{*}d_{k}>0, $$

where $\sigma_{*}\in (0,1)$.

*Case 3:* $\bar{A}_{k}>0$ *.* The proof can be found in [41]

Similar to the proof of Theorem 3.1 in [51], we can establish the global convergence theorem of Algorithm 1. Here, we state the theorem but omit the proof. □

### Theorem 3.1

*Let the conditions of Lemma* 3.3 *hold*; *then we have*

3.3 $$ \liminf_{k\rightarrow \infty } \Vert g_{k}\Vert =0. $$

## Superlinear convergence analysis

Based on Theorem 3.1, we suppose that $x^{*}$ is the limit of the sequence $\{x_{k}\}$. To establish the superlinear convergence of Algorithm 1, the following additional assumption is needed.

### Assumption B

$g(x^{*})=0$ *with* $x_{k}\rightarrow x^{*}$. $G(x^{*}) $ *is positive definite and Hölder continuous at* $x^{*}$, *namely*, *for all* *x* *in the neighborhood of* $x^{*}$, *there exist constants* $u\geq (0,1)$ *and* $\zeta \geq 0$ *satisfying*

4.1 $$ \bigl\Vert G(x)-G\bigl(x^{*}\bigr)\bigr\Vert \le \zeta \bigl\Vert x-x^{*}\bigr\Vert ^{u}, $$

*where* $G(x)=\nabla^{2} f(x)$.

In a way similar to [41], we can obtain the superlinear convergence of Algorithm 1, which we state as follows but we omit its proof.

### Theorem 4.1

*Let Assumption* A *and* B *hold and* $\{x_{k}\}$ *be generated by Algorithm* 1. *Then the sequence* $\{x_{k}\}$ *superlinearly tends to* $x^{*}$.

## Numerical results

This section reports the numerical results of Algorithm 1. All code was written in MATLAB 7.0 and run on a PC with a 2.60 GHz CPU processor, 256 MB memory and the Windows XP operating system. The parameters are chosen as $\delta =0.1$, $\sigma =0.9$, $\varepsilon =10^{-5}$, $\epsilon_{1}=0.1$, $\epsilon_{2}=0.01$, $p=5$, $M_{0}=8$, and the initial matrix $B_{0}=I$ is the unit matrix. Since the line search cannot ensure the descent condition $d_{k}^{T}g_{k}<0$, an uphill search direction may occur in the numerical experiments. In this case, the line search rule may fail. To avoid this case, the step size $\alpha_{k}$ is accepted if the search number is greater than 25 in the line search. The following is the *Himmeblau* stop rule: If $\vert f(x_{k})\vert > e_{1}$, let $\mathit{stop}1=\frac{\vert f(x_{k})-f(x_{k+1})\vert }{\vert f(x_{k})\vert }$; otherwise, let $\mathit{stop}1=\vert f(x_{k})-f(x_{k+1})\vert $. In the experiment, if $\Vert g(x)\Vert < \varepsilon $ or $\mathit{stop} 1 < e_{2}$ satisfies $e_{1}=e _{2}=10^{-5}$, we end the program.

### [57] problems

It has been proved that [57] problems with initial points are an effective tool to estimate the performance of algorithms and are one of the most commonly used sets of optimization problems. Many scholars use these problems to assess their algorithms (see [23, 40, 42, 51]). In this paper, we also perform experiments on these problems. The detailed numerical results are listed in Table 1, where the columns of Table 1 have the following meaning:

Problem: the name of the test problem;
Dim: the dimensions of the problem;
NI: the total number of iterations;
Time: the cpu time in seconds;
NFG: $NFG=NF+5NG$, where *NF* and *NG* are the total number of function and gradient evaluations, respectively (see [47]).

**Table 1:** **Numerical results**

**Problem**	**Dim**	**BFGS-WP NI/NFG/Time**	**BFGS-WP-Zhang NI/NFG/Time**	**BFGS-Non NI/NFG/Time**	**BFGS-M-Non NI/NFG/Time**
ROSE	2	35/590/4.506480e−002	31/611/4.882020e−002	2/19/6.259000e−003	2/19/6.259000e−003
FROTH	2	9/116/1.376980e−002	7/90/1.001440e−002	2/19/6.259000e−003	2/19/7.510800e−003
BADSCP	2	43/706/5.507920e−002	43/706/5.507920e−002	8/264/2.753960e−002	8/264/2.753960e−002
BADSCB	2	3/60/1.126620e−002	3/60/1.001440e−002	3/32/7.510800e−003	3/32/6.259000e−003
BEALE	2	15/220/2.128060e−002	16/226/2.002880e−002	2/19/6.259000e−003	2/19/6.259000e−003
JENSAM	2	2/42/1.126620e−002	2/42/1.001440e−002	2/19/6.259000e−003	2/19/8.762600e−003
HELIX	3	34/483/4.381300e−002	23/325/3.004320e−002	169/2,191/2.090506e−001	87/1,163/1.114102e−001
BARD	3	16/229/3.004320e−002	14/182/2.503600e−002	72/930/1.226764e−001	72/930/1.226764e−001
GAUSS	3	2/19/6.259000e−003	2/19/6.259000e−003	2/19/7.510800e−003	2/19/7.510800e−003
MEYER	3	2/42/1.376980e−002	2/42/1.251800e−002	2/32/1.126620e−002	2/32/1.251800e−002
GULF	3	2/42/1.502160e−002	2/42/1.502160e−002	2/19/3.755400e−003	2/19/1.001440e−002
BOX	3	2/42/1.251800e−002	2/42/1.126620e−002	2/19/7.510800e−003	2/19/8.762600e−003
SING	4	20/280/2.503600e−002	18/269/2.503600e−002	2/19/6.259000e−003	2/19/7.510800e−003
WOOD	4	19/271/2.628780e−002	20/289/2.753960e−002	2/19/6.259000e−003	2/19/6.259000e−003
KOWOSB	4	21/295/3.505040e−002	23/324/3.630220e−002	83/1,077/1.314390e−001	104/1,345/1.664894e−001
BD	4	17/244/3.505040e−002	19/276/3.880580e−002	2/19/7.510800e−003	2/19/1.001440e−002
OSB1	5	2/42/2.128060e−002	2/42/1.877700e−002	2/19/7.510800e−003	2/19/1.001440e−002
BIGGS	6	25/322/4.506480e−002	7/108/2.253240e−002	15/330/4.381300e−002	21/287/4.130940e−002
OSB2	11	3/56/6.259000e−002	3/56/6.259000e−002	3/33/1.877700e−002	3/33/2.002880e−002
WATSON	20	31/457/3.880580e−001	29/412/3.555112e−001	2/19/2.002880e−002	2/19/2.253240e−002
ROSEX	100	229/3,704/1.268073e+000	276/4,359/1.512174e+000	2/19/1.126620e−002	2/19/1.251800e−002
SINGX	400	65/922/1.174939e+001	155/2,375/2.844465e+001	2/19/2.065470e−001	2/19/2.115542e−001
PEN1	400	2/47/7.247922e−001	2/47/7.310512e−001	2/19/1.940290e−001	2/19/1.927772e−001
PEN2	200	2/25/6.884900e−002	2/25/6.634540e−002	2/19/6.008640e−002	2/19/6.384180e−002
VARDIM	100	2/47/2.879140e−002	2/47/2.879140e−002	2/19/1.001440e−002	2/19/8.762600e−003
TRIG	500	9/138/1.627340e+002	9/144/1.671604e+002	8/146/1.700345e+002	50/876/1.039274e+003
BV	500	2/19/3.492522e−001	2/19/3.492522e−001	2/19/3.480004e−001	2/19/3.517558e−001
IE	500	6/71/7.711088e+000	6/71/7.706081e+000	6/71/7.722354e+000	6/71/7.772426e+000
TRID	500	53/760/1.622333e+001	50/727/1.501159e+001	564/7,325/1.690631e+002	564/7,325/1.692333e+002
BAND	500	12/275/5.551733e+000	12/238/4.696754e+000	2/19/4.781876e−001	2/19/4.431372e−001
LIN	500	2/19/4.719286e−001	2/19/4.744322e−001	2/19/4.806912e−001	2/19/4.719286e−001
LIN1	500	3/32/9.363464e−001	3/32/9.388500e−001	3/31/9.050514e−001	3/31/9.025478e−001
LIN0	500	3/32/1.165426e+000	3/32/1.161670e+000	3/31/1.119109e+000	3/31/1.130375e+000

In Table 1, ‘BFGS-WP’, ‘BFGS-Non’, ‘BFGS-WP-Zhang’, and ‘BFGS-M-Non’ stand for the normal BFGS formula with WWP rule, the normal BFGS formula with GLL rule, the modified BFGS equation (1.10) with WWP rule, and MN-BFGS-A, respectively. The numerical results in Table 1 indicate that the proposed method is competitive with the other three similar methods.

**Figure 1:**
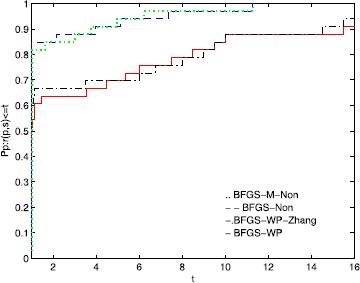
**Performance profiles of these methods (NI).**

**Figure 2:**
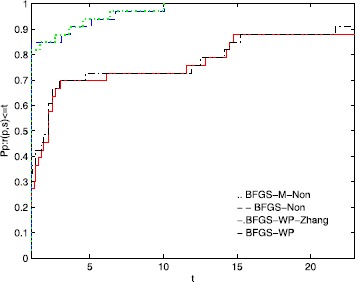
**Performance profiles of these methods (NFG).**

**Figure 3:**
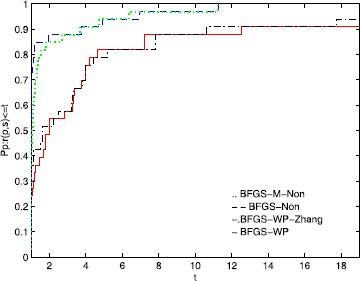
**Performance profiles of these methods (Time).**

To directly illustrate the performance of these methods, we utilize the tool of Dolan and Moré [58] to analyze their efficiency. Figures 1, 2, and 3 show that the performance is related to *NI*, *NFG*, and *Time*, respectively. According to these three figures, the MN-BFGS-A method has the best performance (the highest probability of being the optimal solver).

Figure 1 shows that BFGS-M-Non and BFGS-Non outperform BFGS-WP and BFGS-WP-Zhang on approximately 9% and 6% of the problems, respectively. The BFGS-WP-Zhang and BFGS-WP methods can successfully solve 94% and 91% of the test problems, respectively.

Figure 2 shows that BFGS-M-Non and BFGS-Non are superior to BFGS-WP and BFGS-WP-Zhang on approximately 12% and 9% of these problems, respectively. The BFGS-M-Non and BFGS-Non methods solve 100% of the test problems at $t\approx 10$. The BFGS-WP-Zhang and the BFGS-WP methods solve the test problems with probabilities of 91% and 88%, respectively.

Figure 3 shows that the success rates when using the BFGS-M-Non and BFGS-Non methods to address the test problems are higher than the success rates when using BFGS-WP and BFGS-WP-Zhang by approximately 6% and 9%, respectively. Additionally, the BFGS-M-Non and BFGS-Non algorithms can address almost all the test problems. Moreover, BFGS-WP-Zhang has better results than BFGS-WP.

### Benchmark problems

The benchmark problems listed in Table 2 are widely applied in various practical engineering situations. A function is multimodal if it has two or more local optima. A function *p* of the responding variables is separable provided that it can be rewritten as a sum of *p* functions of just one variable [59]. Separability is closely related to the concept of epistasis or interrelation among the variables of a function. Non-separable functions are more difficult to optimize because the accuracy of the searching direction depends on two or more variables. By contrast, separable functions can be optimized for each variable in turn. The problem is even more difficult if the function is multimodal. The search process must be able to avoid the regions around local minima in order to approximate, as closely as possible, the global optimum. The most complex case appears when the local optima are randomly distributed in the search space.

**Table 2:** **Definition of the benchmark problems and their features**

**Function**	**Definition**	**Multimodal?**	**Separable?**	**Regular?**
Sphere	$f_{Sph}(x)=\sum_{i=1}^{p}x_{i}^{2}$	no	yes	n/a
	$x_{i}\in [-5.12,5.12]$, $x^{*}=(0,0,\ldots,0)$, $f_{Sph}(x^{*})=0$.			
Schwefel’s	$f_{SchDS}(x)=\sum_{i=1}^{p}(\sum_{j=1}^{i}x_{j})^{2}$	no	no	n/a
	$x_{i}\in [-65.536,65.536]$, $x^{*}=(0,0,\ldots,0)$, $f_{SchDS}(x^{*})=0$.			
Griewank	$f_{Gri}(x)=1+\sum_{i=1}^{p}\frac{x_{i}^{2}}{4{,}000}-\prod_{i=1}^{p}\cos \frac{x_{i}}{i}$	yes	no	yes
	$x_{i}\in [-600,600]$, $x^{*}=(0,0,\ldots,0)$, $f_{Gri}(x^{*})=0$.			
Rosenbrock	$f_{Ros}(x)=\sum_{i=1}^{p}[100(x_{i+1}-x_{i}^{2})^{2}+(x_{i}-1)^{2}]$	no	no	n/a
	$x_{i}\in [-2.048,2.048]$, $x^{*}=(1,1,\ldots,1)$, $f_{Ros}(x^{*})=0$.			
Ackley	$f_{Ack}(x)=20+e-20 e^{-0.2\sqrt{\frac{1}{p}\sum _{i=1}^{p}x_{i}^{2}}}-e^{\frac{1}{p}\sum _{i=1}^{p}\cos (2\pi x_{i})}$	yes	no	yes
	$x_{i}\in [-30,30]$, $x^{*}=(0,0,\ldots,0)$, $f_{Ack}(x^{*})=0$.			

The dimensionality of the search space is another important factor in the complexity of the problem. A study of the dimensionality problem and its features was conducted by Friedman [60]. To establish the same degree of difficulty in all cases, a search space of dimensionality $p=30$ is chosen for all the functions. In the experiment, we do not fix the value to $p=30$, namely, it can be larger than 30. The exact dimensions can be found in Table 3.

**Table 3:** **Numerical results of the benchmark problems**

**Problem/** $\boldsymbol{x_{0}}$	**Dim**	**BFGS-WP NI/NFG/Time**	**BFGS-WP-Zhang NI/NFG/Time**	**BFGS-Non NI/NFG/Time**	**BFGS-M-Non NI/NFG/Time**
Sphere/$x_{Sph10}$	30	2/19/1.562500e−001	2/19/1.562500e−002	2/19/4.687500e−002	2/19/4.687500e−002
	500	2/19/2.031250e−001	2/19/3.125000e−001	2/19/2.656250e−001	2/19/2.187500e−001
	1,000	2/19/1.015625e+000	2/19/1.093750e+000	2/19/1.062500e+000	2/19/1.046875e+000
Sphere/$x_{Sph20}$	30	2/19/0	2/19/0	2/19/0	2/19/0
	500	2/19/1.875000e−001	2/19/2.500000e−001	2/19/2.187500e−001	2/19/1.875000e−001
	1,000	2/19/9.531250e−001	2/19/1.046875e+000	2/19/1.031250e+000	2/19/1.218750e+000
Sphere/$x_{Sph30}$	30	2/19/0	2/19/0	2/19/0	2/19/0
	500	2/19/2.031250e−001	2/19/2.812500e−001	2/19/2.343750e−001	2/19/1.718750e−001
	1,000	2/19/1.015625e+000	2/19/9.687500e−001	2/19/9.531250e−001	2/19/9.843750e−001
Sphere/$x_{Sph40}$	30	2/19/0	2/19/0	2/19/0	2/19/0
	500	2/19/1.718750e−001	2/19/2.343750e−001	2/19/2.187500e−001	2/19/1.250000e−001
	1,000	2/19/9.218750e−001	2/19/1	2/19/1	2/19/1.015625e+000
Schwefel’s/$x_{SchDs10}$	30	3/32/0	3/32/6.250000e−002	3/32/6.250000e−002	3/32/0
	50	3/32/0	3/32/0	3/32/6.250000e−002	3/32/6.250000e−002
	100	4/45/1.562500e−001	4/45/2.500000e−001	6/70/3.750000e−001	6/70/4.062500e−001
Schwefel’s/$x_{SchDs20}$	30	2/19/6.250000e−002	2/19/0	2/19/0	2/19/0
	50	2/19/0	2/19/6.250000e−002	2/19/0	2/19/0
	100	3/32/1.875000e−001	3/32/1.250000e−001	3/32/1.875000e−001	3/32/1.718750e−001
Schwefel’s/$x_{SchDs30}$	30	3/32/0	3/32/6.250000e−002	3/32/0	3/32/0
	50	3/32/6.250000e−002	3/32/0	3/32/0	3/32/6.250000e−002
	100	3/32/1.875000e−001	3/32/1.250000e−001	3/32/1.875000e−001	3/32/1.250000e−001
Schwefel’s/$x_{SchDs40}$	30	2/19/0	2/19/0	2/19/0	2/19/0
	50	2/19/0	2/19/6.250000e−002	2/19/0	2/19/0
	100	2/19/6.250000e−002	2/19/6.250000e−002	2/19/1.250000e−001	2/19/6.250000e−002
Griewank/$x_{Gri10}$	30	3/37/0	3/37/0	11/258/6.250000e−002	9/130/6.250000e−002
	500	2/24/5.781250e−001	2/24/5.312500e−001	2/24/5.781250e−001	2/24/6.406250e−001
	1,000	2/24/1.984375e+000	2/24/1.656250e+000	2/24/1.671875e+000	2/24/1.625000e+000
Griewank/$x_{Gri20}$	30	4/75/0	4/75/4.687500e−002	4/59/0	4/58/0
	500	2/24/6.718750e−001	2/24/3.437500e−001	2/24/4.062500e−001	2/24/6.562500e−001
	1,000	2/24/1.765625e+000	2/24/1.796875e+000	2/24/1.859375e+000	2/24/1.640625e+000
Griewank/$x_{Gri30}$	30	3/38/0	3/37/4.687500e−002	11/394/1.250000e−001	9/178/0
	500	2/24/5.625000e−001	2/24/5.468750e−001	2/24/5.625000e−001	2/24/5.781250e−001
	1,000	2/24/2.046875e+000	2/24/1.531250e+000	2/24/1.468750e+000	2/24/1.421875e+000
Griewank/$x_{Gri40}$	30	15/200/6.250000e−002	19/249/6.250000e−002	9/502/6.250000e−002	18/446/1.250000e−001
	500	2/24/6.093750e−001	2/24/2.968750e−001	2/24/5.468750e−001	2/24/5.468750e−001
	1,000	2/24/1.843750e+000	2/24/1.468750e+000	2/24/1.828125e+000	2/24/1.781250e+000
Rosenbrock/$x_{Ros10}$	30	34/483/1.406250e−001	5/116/0	2/19/0	2/19/0
	500	30/419/3.431250e+001	5/116/2.031250e+000	2/19/2.187500e−001	2/19/1.875000e−001
	1,000	28/393/2.136875e+002	6/152/2.207813e+001	2/19/1.078125e+000	2/19/9.375000e−001
Rosenbrock/$x_{Ros20}$	30	30/467/9.375000e−002	5/121/0	2/19/0	2/19/0
	500	16/268/1.650000e+001	3/38/6.250000e−001	2/19/1.875000e−001	2/19/2.187500e−001
	1,000	17/286/1.181094e+002	3/38/3.453125e+000	2/19/1.062500e+000	2/19/9.062500e−001
Rosenbrock/$x_{Ros30}$	30	8/134/0	7/141/0	2/19/0	2/19/0
	500	9/154/6.828125e+000	6/110/3.546875e+000	2/19/2.031250e−001	2/19/2.187500e−001
	1,000	7/115/3.090625e+001	5/92/1.373438e+001	2/19/1.125000e+000	2/19/1.156250e+000
Rosenbrock/$x_{Ros40}$	30	8/140/0	5/102/0	2/19/6.250000e−002	2/19/0
	500	12/186/1.185938e+001	6/105/5.203125e+000	2/19/2.343750e−001	2/19/2.031250e−001
	1,000	15/226/101	6/105/2.275000e+001	2/19/1.062500e+000	2/19/1.015625e+000
Ackley/$x_{Ack10}$	30	5/68/6.250000e−002	6/80/0	6/83/0	6/80/0
	500	5/67/2.343750e+000	5/64/1.937500e+000	5/67/2.046875e+000	5/68/2.171875e+000
	1,000	5/66/1.407813e+001	6/79/2.229688e+001	5/66/1.410938e+001	6/79/2.278125e+001
Ackley/$x_{Ack20}$	30	2/42/0	2/42/0	7/99/6.250000e−002	7/97/6.250000e−002
	500	6/79/3.250000e+000	6/77/3.640625e+000	6/79/3.671875e+000	6/77/3.593750e+000
	1,000	5/66/1.354688e+001	5/63/1.443750e+001	5/65/1.423438e+001	5/66/1.429688e+001
Ackley/$x_{Ack30}$	30	9/126/0	5/67/0	9/126/6.250000e−002	6/83/0
	500	6/88/3.500000e+000	4/50/1.187500e+000	6/88/3.437500e+000	6/78/2.828125e+000
	1,000	4/53/7.531250e+000	4/51/7.671875e+000	7/95/3.085938e+001	6/77/2.229688e+001
Ackley/$x_{Ack40}$	30	4/56/6.250000e−002	4/57/6.250000e−002	8/108/0	7/92/4.687500e−002
	500	4/55/1.343750e+000	4/54/1.015625e+000	7/98/4.062500e+000	7/92/4.562500e+000
	1,000	6/84/2.232813e+001	6/79/2.256250e+001	6/84/2.310938e+001	6/77/2.254688e+001
Total CPU Time		516.1562	161.5781	115.0938	115.0156

However, the effectiveness of one algorithm compared another algorithm cannot be determined based on the number of problems that it solves better. The ‘no free lunch’ theorem (see [61]) states that provided we compare two searching algorithms with all possible functions, the performance of any two algorithms will be, on average, the same. As a result, attempting to find a perfect test set where all the functions are present to determine whether an algorithm is better than another algorithm for every function is a fruitless task. Therefore, when an algorithm is evaluated, we identify the types of problems where its performance is good to characterize the types of problems for which the algorithm is suitable. The authors previously studied functions to be optimized to construct a test set with a better selection of fewer functions (see [62, 63]). This enables us to draw conclusions about the performance of the algorithm depending on the type of function.

The above benchmark problems and the discussions of the choice of test problems for an algorithm can be found at

- http://www.cs.cmu.edu/afs/cs/project/jair/pub/volume24/ortizboyer05a-html/node6.html.

Many scholars use these problems to test numerical optimization methods (see [64, 65] etc.). Based on the above discussions, in this subsection, we test the four algorithms on the Benchmark problems. The test results are presented in Table 3, where $x_{0}$ denotes the initial point, $x_{Sph10}=(-2,-2,\ldots,-2)$, $x_{Sph20}=(2,2,\ldots,2)$, $x_{Sph30}=(-2,0,-2,0,\ldots)$, $x_{Sph40}=(2,0,2,0,\ldots)$, $x_{SchDS10}=(-0.0001,-0.0001,\ldots,-0.0001)$, $x_{SchDS20}=(0.00001,0.00001,\ldots, 0.00001)$, $x_{SchDS30}=(-0.0001,0,-0.0001,0, \ldots)$, $x_{SchDS40}=(0.00001,0,0.00001,0,\ldots)$, $x_{Gri10}=(-21,-21,\ldots,-21)$, $x_{Gri20}=(32,32,\ldots,32)$, $x_{Gri30}=(-21,0,-21,0,\ldots)$, $x_{Gri40}=(32,0,32,0,\ldots)$, $x_{Ros10}=(1.45,1.45,\ldots,1.45)$, $x_{Ros20}=(2.1,2.1,\ldots,2.1)$, $x_{Ros30}=(1.45,0,1.45, 0,\ldots)$, $x_{Ros40}=(2.1,0,2.1,0,\ldots)$, $x_{Ack10}=(-0.002,-0.002,\ldots,-0.002)$, $x_{Ack20}=(0.004, 0.004,\ldots,0.004)$, $x_{Ack30}=(-0.002,0,-0.002,0,\ldots)$, and $x_{Ack40}=(0.004,0,0.004,0,\ldots)$.

**Figure 4:**
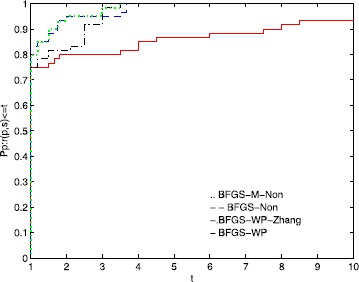
**Performance profiles of these methods (NI).**

**Figure 5:**
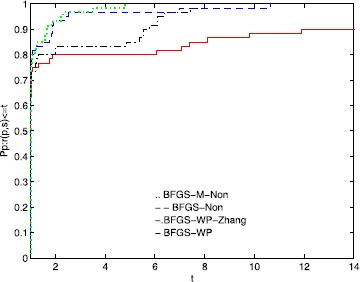
**Performance profiles of these methods (NFG).**

The numerical results in Table 3 show that the proposed algorithm performs the best among the four methods. The total cpu time of the proposed algorithm is the shortest. BFGS-Non performs better than BFGS-WP and BFGS-WP-Zhang, which is consistent with the results of [51]. Additionally, BFGS-WP-Zhang performs better than BFGS-WP, which is consistent with the results of [42]. To directly illustrate the performances of these four methods, we also use the tool of Dolan and Moré [58] to analyze the results with respect to NI and NFG in Table 3. Figures 4 and 5 show their performances.

Figure 4 indicates that BFGS-WP can solve approximately 93% of the test problems and that the other three methods can solve all the problems. The proposed algorithm solves the problems in the shortest amount of time.

The performance in Figure 5 is similar to that in Figure 4. BFGS-WP can solve approximately 95% of the test problems, while the other methods can solve all the problems.

According to these two figures, the proposed algorithm has the best performance among these four methods, and the BFGS-WP performs the worst. In summary, based on the numerical results of the [57] and benchmark problems, the GLL nonmonotone line search with quasi-Newton update is more effective than the normal WWP line search with quasi-Newton update, which is consistent with the results of [47, 51]. Moreover, these numerical results indicate that the modified BFGS equation (1.10) is better than the normal BFGS update, which is consistent with the results of [42]. Furthermore, the proposed algorithm is competitive with the related methods.

## Conclusion

(i) This paper conducts a further study of the modified BFGS update formula in [43]. The main contribution is the global convergence and superlinear convergence for generally convex functions. The numerical results show that the proposed method is competitive with other quasi-Newton methods for the test problems.
(ii) In contrast to [42] and [43], this paper achieves both superlinear and global convergence. Moreover, the convergence is obtained for generally convex functions, whereas the other two papers only obtained convergence for uniformly convex functions. The conditions of this paper are weaker than those of the previous research.
(iii) For further research, we should study the performance of the new algorithm under different stop rules and in different testing environments (such as [66]). Moreover, more numerical experiments for large practical problems should be performed in the future.
